# Caffeine, a Risk Factor for Osteoarthritis and Longitudinal Bone Growth Inhibition

**DOI:** 10.3390/jcm9041163

**Published:** 2020-04-18

**Authors:** María Guillán-Fresco, Eloi Franco-Trepat, Ana Alonso-Pérez, Alberto Jorge-Mora, Miriam López-Fagúndez, Andrés Pazos-Pérez, Oreste Gualillo, Rodolfo Gómez

**Affiliations:** 1 Musculoskeletal Pathology Group, Institute IDIS, Santiago University Clinical Hospital, SERGAS, 15706 Santiago de Compostela, Spain; maria.guillan.fresco@sergas.es (M.G.-F.); eloi.franco.trepat@sergas.es (E.F.-T.); ana.alonso.perez@sergas.es (A.A.-P.); alberto.agustin.jorge.mora@sergas.es (A.J.-M.); miriam.lopez.fagundez@sergas.es (M.L.-F.); andrespazosperez@gmail.com (A.P.-P.); 2 Research Laboratory 9, Institute of Medical Research, SERGAS, Santiago University Clinical Hospital, 15706 Santiago de Compostela, Spain; oreste.gualillo@sergas.es

**Keywords:** osteoarthritis, articular cartilage, growth plate cartilage, catabolism, long bone growth inhibition, growth retardation, caffeine

## Abstract

Osteoarthritis (OA), the most common chronic rheumatic disease, is mainly characterized by a progressive degradation of the hyaline articular cartilage, which is essential for correct joint function, lubrication, and resistance. Articular cartilage disturbances lead to joint failure, pain, and disability. Hyaline cartilage is also present in the growth plate and plays a key role in longitudinal bone growth. Alterations of this cartilage by diverse pathologies have been related to longitudinal bone growth inhibition (LBGI), which leads to growth retardation. Diet can play a crucial role in processes involved in the OA and LBGI’s onset and evolution. Specifically, there is ample evidence pointing to the negative impacts of caffeine consumption on hyaline cartilage. However, its effects on these tissues have not been reviewed. Accordingly, in this review, we summarize all current knowledge in the PubMed database about caffeine catabolic effects on articular and growth plate cartilage. Specifically, we focus on the correlation between OA and LBGI with caffeine prenatal or direct exposure. Overall, there is ample evidence indicating that caffeine intake negatively affects the physiology of both articular and growth plate cartilage, increasing consumers predisposition to suffer OA and LBGI. As a result, caffeine consumption should be avoided for these pathologies.

## 1. Introduction

Osteoarthritis (OA) is the most common worldwide chronic rheumatic disease and the main culprit of disability among the middle-aged and elderly [1,2,3,4,5,6,7,8]. It is characterized by progressive articular cartilage degradation. However, the whole joint is normally compromised [2,3,4,5,6,7,8]. Articular cartilage is a type of hyaline cartilage that covers joint surfaces and is involved in maintaining the correct function of diarthrodial joints, lubrication, and resistance to mechanical loading [5,6,7,8]. As a result of OA alterations, symptoms like pain, stiffness, and loss of function occur, leading to an increase of personal dependency [1,8,9]. It is estimated that 250 million people have knee OA, which is linked to important economic costs [1].

Hyaline cartilage is also found in the embryonic stages of endochondral bones and in the growth plate, where it plays a crucial role in the longitudinal growth of long bones. Moreover, it can also be found during the healing process of broken bones [7,8,10]. Alteration of the hyaline cartilage of the growth plate promotes longitudinal bone growth inhibition (LBGI) and leads to growth retardation [7,11,12]. Among the diverse pathologies that affect this type of cartilage, chronic inflammatory diseases stand out. These diseases have a negative impact on bone development, with a prevalence of bone alterations between 17% and 40% [7,11,12].

Multiple reports have exhibited caffeine’s harmful effects on hyaline cartilage. Therefore, considering the elevated worldwide consumption of caffeine-rich products, in this review, we summarize all the relevant information on the role of caffeine in the physiology and pathophysiology of articular and growth plate cartilage. Additionally, we also address its role as a potential environmental hazard linked to OA and LBGI.

## 2. Osteoarthritis: Articular Cartilage Catabolism

**Figure 1:**
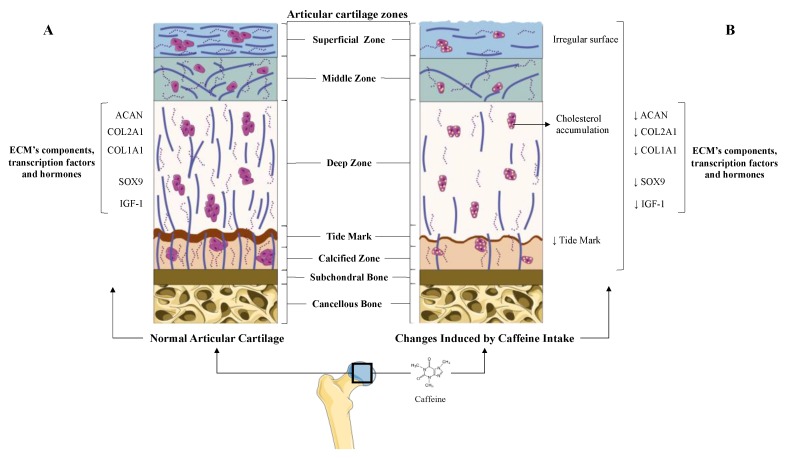
Comparison between healthy articular cartilage and the changes induced on it by caffeine. (**A**) Normal articular cartilage appearance. Articular cartilage is composed of chondrocytes and its extracellular matrix (ECM) components chondrocytes are crucial for the maintenance and repair of the ECM. They respond to a variety of stimuli, such as cytokines, mechanical loading, and growth factors. Among these, insulin growth factor 1 (IGF-1) and Transforming Growth Factor Beta 1 (TGF-ß1) are involved in cartilage homeostasis and chondrocyte responses to mechanical loading. Likewise, in healthy conditions, the articular cartilage expresses high levels of ECM components to guarantee the chondrocyte’s viability and proliferation. Additionally, a wide and remarkably tidemark is observed, as well as a regular surface that provides the ideal biomechanical properties to the joint. (**B**) The articular cartilage changes induced by caffeine intake. Caffeine consumption has been associated with several alterations in articular cartilage, similar to those that appear in osteoarthritis (OA). This alkaloid reduces the synthesis of major cartilage ECM components. It also diminishes chondrocyte proliferation, decreases the tidemark, and is associated with an irregular surface of the superficial zone of the cartilage. Additionally, caffeine is linked to lower chondrocyte quality due to cholesterol accumulation.

Articular cartilage is a specialized type of hyaline cartilage that covers the contact surfaces of diarthrodial joints [5,6]. This tissue is devoid of nerves, blood, and lymphatic vessels, which hampers its capacity to be repaired [5,13]. Articular cartilage is composed of resting chondrocytes and the extracellular matrix (ECM), which is mainly composed of water, collagens (Collagen type I alpha 1 (COL1A1), Collagen type II alpha 1 (COL2A1), etc.), and proteoglycans (agreccan (ACAN)) [5,6,13]. The specific composition of the ECM of the cartilage is essential for the maintenance of its unique mechanical properties and is indispensable for guaranteeing chondrocyte survival [5,6,13] (Figure 1).

Articular cartilage destruction in OA has been evidenced by joint space narrowing [2,3,4]. Although the etiology of OA is not fully understood, it is well-known that OA chondrocyte phenotypic changes that affect ECM composition promote cartilage degradation [8,9]. One of these changes involve the acquisition of a hypertrophic-like phenotype [8,9], which resembles the differentiation process of growth plate chondrocytes (GPChs) [8]. In physiological conditions, articular chondrocytes exist in a resting state and do not reach terminal differentiation [8,9]. However, OA chondrocytes express specific hypertrophic markers, such as collagen type X (COLX), matrix metalloproteinase 13 (MMP13), osteopontin (SPP1, also known as OPN), osteocalcin (OCN), osteonectin (SPARC), Runt-related transcription factor 2 (Runx2), Vascular Endothelial Growth Factor (VEGF), and alkaline phosphatase (ALP) [8]. At the same time, OA chondrocytes also present lower expression levels of the SRY-Box Transcription factor 9 (Sox9) [8]. As a result, all these changes contribute to ECM degradation, thereby affecting the synthesis of ACAN and COL2A1, two of the main components of the ECM.

There are several risk factors for OA development, such as certain genetic profiles, gender, aging, ethnicity, exercise, metabolic alterations, alcohol consumption, obesity, coffee intake, and diet [2,3,4,14]. Notably, multiple modifiable risk factors are closely related to dietary habits, which suggests that diet could play a key role in OA pathogeny.

## 3. Longitudinal Bone Growth Inhibition

**Figure 2:**
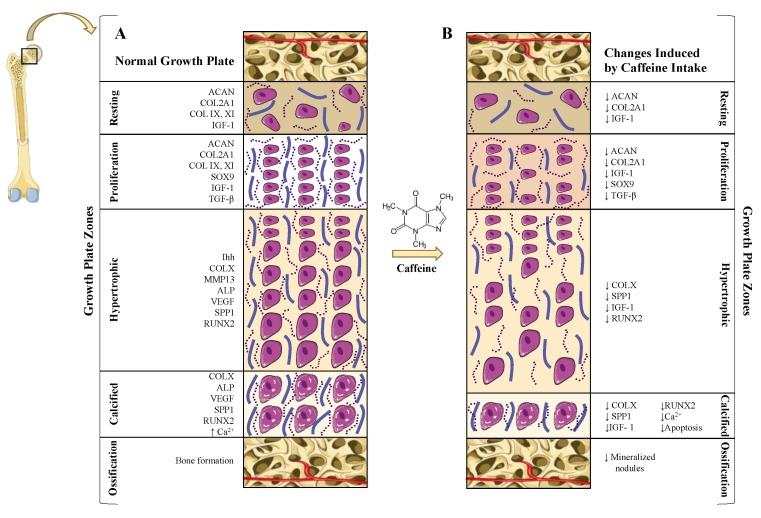
Comparison between normal growth plate cartilage and the changes induced by caffeine. (**A**) Normal growth plate cartilage. The growth plate cartilage is organized in different zones: the resting zone; the proliferation zone, characterized by packed cell columns; the hypertrophic zone (HZ), where cells increase their volume and promote Extracellular Matrix (ECM) production; and the calcified zone, where chondrocytes die, and the ECM is mineralized and remodeled to allow the invasion of bone remodeling cells and blood vessels. The process by which chondrocytes differentiate to give room to bone is called endochondral ossification (EO). Among the factors that modulate GPCh physiology, growth hormone (GH), Insulin Growth Factor 1 (IGF-1), and Transforming Growth Factor ß1 (TGF-ß1) stand out. Likewise, this differentiation process and matrix production are controlled by key transcription factors, such as Sox9 and Runx2. The metalloproteinase MMP13 plays a key role in matrix remodeling. (**B**) Changes in growth plate cartilage induced by caffeine intake. Caffeine consumption has been related to growth retardation due to its impact on the growth plate cartilage. It has a negative impact on the main components of the ECM, as well as in some crucial transcription factors involved in chondrocyte differentiation. Caffeine also interacts with the GH/IGF-1 axis, leading to a reduction in IGF-1 signaling. Additionally, caffeine decreases intracellular calcium concentration (Ca^2+^), as well as chondrocyte apoptosis. Among the morphologic changes induced by caffeine intake, a wider hypertrophic zone and a reduction of mineralization stand out.

Longitudinal bone growth is a process that takes places from the fetal period to adolescence [12,15]. This growth is mediated by chondrocyte proliferation, condensation, and differentiation in the growth plate during a process called endochondral ossification (EO) [9,10,12,16,17] (Figure 2). Interestingly, a similar process has also been observed in certain types of bone fracture healing [9].

The process of longitudinal bone growth is modulated by the same physiological and pathological factors that regulate the articular cartilage [12]. Likewise, several pathological conditions and pharmacological treatments, such as inflammatory diseases and glucocorticoids (GC) therapy, also affect longitudinal bone growth and have been linked to growth retardation [11,12,15,18,19,20,21,22].

Growth retardation is one of the main complications among children who suffer from chronic inflammatory diseases [12,23]. The complications derived from a long-term inflammatory state on bone growth are not limited to growth retardation. Other alterations, such as short stature, growth disorders, and bone dysmetria, have also been associated with long-term inflammation [12]. Supporting this association, certain proinflammatory cytokines exert a negative impact on growth plate development, resulting in growth failure [12,18,19,22]. Specifically, cytokines like IL-6, TNF-*α*, and IL-1*β* interfere with local and systemic IGF-1 metabolism, reducing its levels and anabolic actions [12,18,19,22]. Accordingly, in vitro studies have determined that some of these cytokines directly inhibit growth plate cartilage anabolism (proteoglycans and collagens synthesis), chondrocyte differentiation, and induced chondrocyte apoptosis [12,18,19,22]. Additionally, it has also been suggested that proinflammatory cytokines may interfere in the steroidogenesis process of the growth plate and, consequently, could exert a negative impact on bone growth [12,22].

Systemic inflammatory diseases are usually treated with GC [15,18,21]. These drugs reduce inflammation, but they also exhibit detrimental effects on the growth plate and even contribute to cytokine-mediated growth retardation [15]. The underlying mechanisms of GC-mediated growth retardation can be indirect, through the reduction of systemic IGF-1 levels and the alteration of calcium (Ca^2+^) metabolism [19,20,21,23]. Nonetheless, GC may also exert direct actions on the growth plate. In vitro studies have shown that chondrocytes exposed to GCs feature reduced proliferation, a reversible and prolonged resting period, a marked reduction in matrix synthesis, greater rates of apoptosis, and reduced ALP activity [18,21].

## 4. Caffeine

Caffeine (1,3,7-trimethylxanthine) is a natural alkaloid belonging to the family of methylxantines [23,24,25,26,27,28,29,30,31]. The main caffeine sources are tea leaves, cola nuts, and coffee and cocoa beans [23,24,25,26,27,28,29,30,31,32].

**Figure 3:**
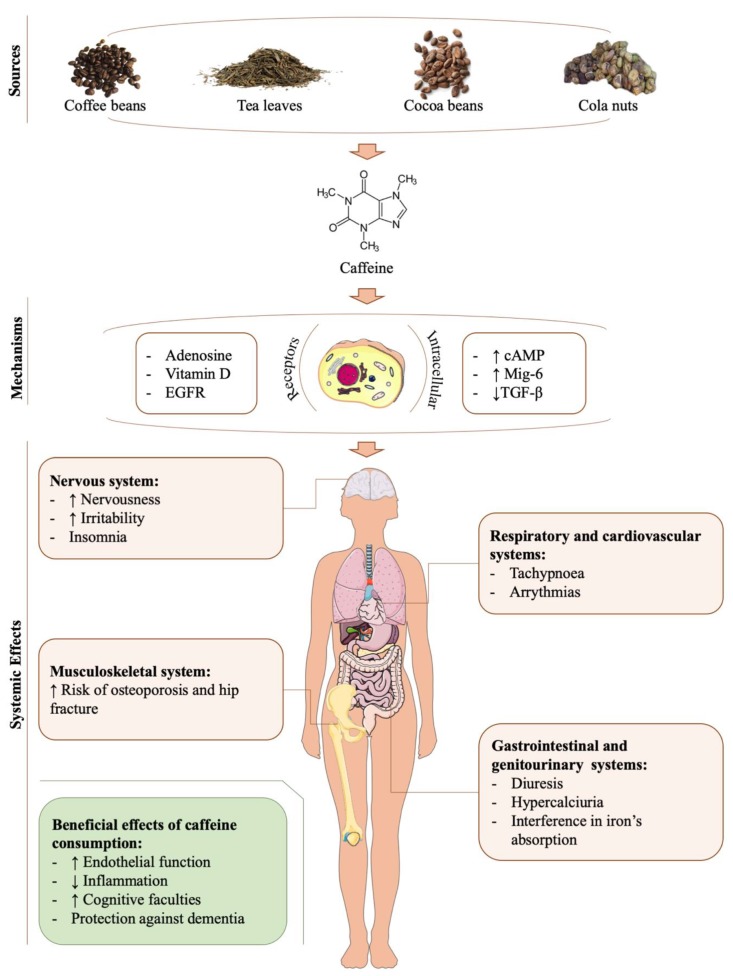
Sources, mechanisms, and systemic effects of caffeine. Caffeine is a natural methylxantine present in coffee and cocoa beans, tea leaves, and cola nuts. It is found in different products, such as beverages (e.g., coffee, tea, soft, or energy drinks) and food (e.g., cocoa, chocolate). Once caffeine is consumed, it exerts its effects through several receptors and some intracellular mediators. The biological effects of caffeine are heterogeneous. Among the most significant of its beneficial activities are improvements in endothelial function, inflammatory processes, and cognitive faculties, as well as protection against dementia. On the other hand, caffeine can have negative systemic effects including alterations in the nervous, respiratory, cardiovascular, gastrointestinal, genitourinary, and musculoskeletal systems.

Caffeine does not have nutritional value. Nonetheless, it is among the most frequently consumed substances, with an average daily ingestion of 120 mg [23,24,25,26,27,28,29,30,31,32]. It is present in beverages (e.g., coffee, tea, soft drinks, and energy drinks), food (e.g., cocoa and chocolate) [23,24,25,26,27,28,29,30,31,32], and some stimulants and is used as an adjuvant to increase the absorption of some medications [23,24,25,26,27,28,29,30,31]. Studies show that coffee (60%–75%) and tea (15%–30%) are the main sources of caffeine for adults, while among children, chocolate and soft drinks are the major intake sources [30,31,32] (Figure 3).

Following its ingestion, caffeine is rapidly absorbed from the gastrointestinal tract into the blood, reaching its maximum blood concentration after 1–1.5 h [30]. Then, caffeine is distributed throughout the whole body and is able to cross the blood–brain barrier and placenta, as well as pass into breast milk and semen [30,32].

The main portion of caffeine metabolism takes place in the liver [30]. Caffeine elimination lasts between 3–7 h, depending on factors such as sex, age, and pregnancy [30,32]. In general, the caffeine half-life in women is 20%–30% shorter than that in men. However, during the first trimester of pregnancy, caffeine’s time of excretion increases from 4 to 18 h. Newborns also exhibit an elevated time of caffeine elimination that ranges from 50 to 100 h. This has been attributed to their deficiency in cytochrome p450, the enzyme that metabolizes caffeine [30,32].

Once caffeine reaches diverse target tissues, it exerts its biological activity through different mechanisms. The most relevant mechanism is adenosine receptor antagonism [30,33]. However, caffeine also interferes with other receptors, such us the Epidermal Growth Factor Receptor (EGFR) [24] and vitamin D receptors [31]. Caffeine also increases intracellular cyclic adenosine monophosphate levels (cAMP) [30]. Moreover, in certain tissues, caffeine increases the mRNA expression of mitogen inducible gene-6 (Mig-6) [24] but decreases TGF-ß mRNA expression [34]. Another important mechanism of action of this alkaloid is that it negatively affects iron absorption but increases the excretion of Ca^2+^ and certain B vitamins [30,31] (Figure 3).

The biological effects of caffeine are heterogeneous (Figure 3). On the one hand, it has been suggested that low doses of caffeine could exert beneficial activities on endothelial function [35,36], in inflammatory processes by reducing some pro-inflammatory cytokines [36,37], and in the improvement of cognitive faculties, as well as providing protection against dementia [35,38]. However, clinical trials conducted in humans have suggested that caffeine is not responsible for the certain beneficial properties observed in caffeinated beverages, like the anti-inflammatory effects of coffee [39,40]. In fact, certain clinical trials have shown that caffeine may enhance post-exercise oxidative stress [41].

On the other hand, evidence has suggested that caffeine, and even coffee [14], may become harmful in a specific concentration. It has been estimated that the threshold of caffeine toxicity is 400 mg/day in healthy adults, 200 mg/day during pregnancy, 100 mg/day in healthy adolescents, and 2.5 mg/kg/day in healthy children [30,32]. It is well-known that in adults, chronic and elevated caffeine intake above 500–600 mg/day (equivalent to four or seven cups of coffee) can cause multiple symptoms, including nervousness, irritability, insomnia, arrhythmias, increased diuresis, tachypnoea, gastrointestinal disturbances, hypercalciuria, female infertility, and an increased risk of osteoporosis and hip fracture [30,31]. In addition to these effects, multiple in vitro and in vivo experiments suggest that caffeine overconsumption could be harmful to the musculoskeletal system, including the hyaline cartilage [23,24,25,26,34,42,43,44] (Figure 3).

## 5. Caffeine’s Role in Cartilage-Related Disorders

The consumption of caffeinated beverages like coffee has been associated with knee OA development in males but not in females [14]. It is known that women have a faster caffeine metabolism [30,32], which in turn could explain this difference between the sexes. Consistent with this, a wide range of studies have suggested the potential harmful effects of caffeine on the musculoskeletal system [23,24,25,26,34,42,43,44]. Specifically, there is a significant bulk of evidence pointing to the role of caffeine in the pathophysiology of both articular and growth plate cartilage [23,24,25,26,34,42,43,44]. Accordingly, caffeine consumption has been associated with severe alterations in the articular cartilage, which have been linked to OA development [23,24,25,26,34,42]. Caffeine has also been related to abnormal bone growth due to alterations in the growth plate cartilage [23,24,25,26,34,42].

### 5.1. Caffeine’s Role in Articular Cartilage: Osteoarthritis

#### 5.1.1. Prenatal Caffeine Exposure

The potential effects of caffeine on the articular cartilage were clearly demonstrated in rat animal models [23,24,25,26,34,42]. In these experiments, prenatal caffeine exposure [23,24,25,26,34,42] (PCE) below the clinical dose of intoxication and in the range of some pregnant women [23,24,25,26,34,42,43,44] significantly affected fetal articular cartilage integrity [23,24,25,26,34,42]. Specifically, histological studies revealed that rat offspring with PCE possess irregular surface cartilage with uneven and altered chondrocytes in the tangential zone [26]. The articular cartilage of these rats also exhibited an irregular and reduced ECM. Interestingly, the tidemark of this articular cartilage was absent, which suggests an altered mineralization process of the hypertrophic chondrocytes [26] (Figure 1). According to all the alterations observed in the histological studies, the Mankin’s score (a scale used for the classification of OA cartilage lesions severity) of the cartilage from the PCE rats was found to be higher than that of their wild type littermates [26]. Underpinning these studies, a molecular analysis of PCE articular cartilage revealed the reduced expression of key components of the ECM, such as COL2A1 and the levels of ACAN and COL1A1 [23,25,26,42]. Likewise, it was also reported that the cartilage of PCE rats exhibited a lower expression of several proteins of the IGF-1 signaling pathway, which suggests reduced anabolic metabolism in this tissue. Among these proteins, the IGF-1 itself, the insulin receptor substrate 1 (IRS-1), and the serine-threonine protein kinase (AKT) were involved in the chondrocytes’ anabolic responses [23,25,26] (Figure 1). Interestingly, the deleterious effects elicited by PCE on the rats’ articular cartilage were still present in adulthood [23,24].

As evidenced above, PCE induces OA-like features in rat cartilage [23]. Similarly, excessive physical activity also promotes cartilage alterations like those found in OA, increasing the risk of OA [23]. In agreement with this, PCE further increased the Mankin’s score of rats exposed to excessive physical activity, suggesting that PCE may also increase OA susceptibility [23]. Supporting this, the cartilage of rats exposed to both PCE and excessive physical activity exhibited rougher articular surfaces, increased worn and torn cartilage surfaces, an increased number of clefts, reduced matrix safranin-O staining (stains proteoglycans and collagens), a reduced number of chondrocytes, and increased blurred tidemarks [23].

Apart from the observed additive effects of PCE-induced cartilage matrix alterations on typical OA matrix lesions, it has been suggested that PCE may also contribute to OA susceptibility through diverse specific mechanisms [23,25,26,42,45]. Among these mechanisms are alterations of chondrocyte vitality and differentiation [42], an alteration of cholesterol metabolism [26], and the reduction of the circulating and local levels of IGF-1 during fetal development [26] (Figure 1).

OA development dramatically affects the chondrocyte phenotype, function, and metabolism, in part reducing the expression of the transcription factor Sox9 [8], which is essential for the preservation of the chondrocyte phenotype and its function [9,10,17]. In this regard, it is noteworthy that caffeine intake in pregnant rats reduced Sox9 expression in the articular chondrocytes of their offspring [42], which is consistent with the reduced vitality and altered differentiation of these cells [42]. This, in turn, involves low-quality articular cartilage [8], which might increase OA susceptibility in adulthood.

Several studies have suggested a relationship between cholesterol accumulation, metabolic syndrome, and the risk of OA [46,47]. Consistent with this, PCE induced low-quality articular cartilage in fetal rats, which was associated with local and systemically altered cholesterol metabolism [26]. On the one hand, PCE reduced the cholesterol efflux in chondrocytes, resulting in a clear accumulation of total cholesterol in these cells [26]. This accumulation was attributed to a lower expression of the IGF1/PI3K/AKT signaling pathway and to a reduced expression of cholesterol efflux genes, such as the Liver X Receptor α (LXRα) and the ATP-binding cassette transporter A1 (ABCA1) [26]. On the other hand, PCE rats, under a high fat diet, exhibited significant hypercholesterolemia, which was suggested to be one of the main causes associated with cholesterol’s influx into the articular cartilage [26,48].

Together with the effects of caffeine on chondrocytes cholesterol metabolism, PCE also reduced the IGF-1 signaling pathway in rat fetal cartilage, maintaining its detrimental effects into adulthood [26]. However, the IGF-1 metabolism alterations by PCE were not limited to the cartilage and were also observed at a systemic level due to a reduction in hepatic IGF-1 production [25,26]. This suggests that low circulating levels of this growth factor might affect the development of articular cartilage. The underlying mechanism of this IGF-1 reduction was attributed to caffeine-induced over-exposure to maternal GC [25]. Accordingly, it has been suggested that PCE could support the existence of a subset of OA with a fetal origin [26].

#### 5.1.2. Direct and Indirect Effect of Caffeine on Articular Chondrocytes

Data from PCE animal studies show that several systemic mechanisms, like the hypercholesterolemia and the reduction of circulating IGF-1 [26], may explain some of caffeine’s modes of action on the articular cartilage. In vitro experiments have revealed that caffeine also exerts direct actions on the chondrocyte’s primary cultures [23,27,33]. Among these activities, caffeine stimulation in a dose-dependent manner was shown to reduce rat chondrocyte proliferation and viability [27]. Similarly, caffeine (1–100 μM) also reduced the mRNA expression of key ECM components (COL2A1 and ACAN) in these cells [23,27]. It also reduced the mRNA expression of several IGF-1 signaling pathway members (IGF1, IGF1-receptor, and AKT) that participate in the chondrocytes’ anabolic responses [23,49] (Figure 1).

One of the mechanisms that has been proposed to explain the direct actions of caffeine on chondrocytes is based on caffeine’s well-known ability to inhibit adenosine receptors [27,33]. The activation of these receptors, expressed in the articular cartilage [27,33], has been described to have anti-inflammatory and anabolic effects on chondrocytes [27,33] and also in other articular cells, including synoviocytes [27,33]. Despite this, the stimulation of equine cartilage explants with caffeine (1 mM) did not block the adenosine-mediated autocrine anabolic responses of the cartilage [33]. Nonetheless, in this experimental setup, theophylline, another methylxanthine, was able to block these actions, suggesting that caffeine could also exert the same effects at higher doses. According to this, and considering the similar phenotypes of articular cartilage chondrocytes and GPChs, it was observed that caffeine stimulation of GPChs reduces the production of ECM components [27].

Finally, the caffeine-mediated imbalance between inflammatory and anti-inflammatory cytokine levels has been proposed to exert a potentially harmful indirect effect on the articular cartilage [45]. In agreement with this, the serum level ratio of the inflammatory cytokine IL-6 and the anti-inflammatory cytokine IL-11 has been proposed as a clinical marker of OA progression [45,50,51]. Interestingly, in vitro experiments of non-articular cells revealed that caffeine reduces the expression of IL-11 [45]; thus, it might increase the IL6/IL11 ratio, which is, in turn, related to an aggressive therapy for OA [45].

### 5.2. Caffeine Effects on the Growth Plate: Longitudinal Bone Growth Inhibition

The physiology and pathophysiology of both articular and growth plate cartilage are tightly related. Indeed, epidemiological studies have underpinned this idea, showing that a lower birth weight (growth retardation) is associated with hand or spine OA [52,53]. As a result, it has been suggested that common intrauterine deleterious factors may support alterations in both tissues [26]. Thus, the PCE, which promotes multiple alterations of the articular cartilage, could also affect the growth plate cartilage.

#### 5.2.1. Prenatal Caffeine Exposure

As it reported for articular cartilage, animal models of PCE also revealed that caffeine exposure during the prenatal stages significantly affects skeletal development and bone growth [23,24,25,26,34,42]. Accordingly, several studies have demonstrated that PCE is related to intrauterine growth retardation (IUGR) in animals [3,13], as well as in humans [43,44]. This alteration is characterized by a low birthweight and shortened bones due to a retarded EO [24,25,26]. Consistent with this, PCE has been associated with significant growth plate abnormalities [23,24,25,26,34,42]. The growth plates of rat PCE fetuses exhibited a reduced number of mineralized nodules, decreased cell viability, lower COL2A1 and ACAN expression [25], and poorer IGF-1 signaling [25]. Moreover, these growth plates presented longer hypertrophic zones (HZ) [24,25,27,42] (Figure 2).

It is well known that exogenous and endogenous GC induces growth retardation [25,54]. In agreement with this, PCE rat models show that the caffeine-mediated over-exposure to maternal GCs is responsible for its deleterious effects on the growth plate [24,33]. In line with this, it was determined that PCE increased the corticosterone level in the blood of PCE rat fetuses [25], which was also associated with a lower IGF-1 signaling pathway in the growth plates of these rats [24,25,26]. In the growth plate, IGF-1 contributes to chondrocyte proliferation, differentiation, and maintenance via the phosphatidylinositol 3-kinase (PI3K)/Akt signaling pathway [23,25]. IGF-1 also plays a critical role in the production of ECM components, such as collagens and proteoglycans (ACAN and COL2A1) [23,25]. As a result, it was suggested that the corticoid-dependent caffeine reduction of the IGF-1 signaling in the growth plate could be responsible for PCE effects on bone growth [23,25,26].

Caffeine’s alteration of GC metabolism may also affect other key factors and signaling pathways involved in the physiology of the growth plate. Among them are the EGFR and the Mig-6 [24,25,26]. It is well known that a loss of EGFR signaling in mice disrupts the growth plate’s normal growth [24,55]. Its deficiency enlarges the growth plate’s HZ, impairs the EO, and leads to notable growth retardation [24,55]. In agreement with this, the suppression of EGFR expression observed in the growth plate HZ of PCE rats was linked to a significantly widened HZ of the growth plate (Figure 2). Supporting this, in the growth plate of these rats, an upregulation was also observed in the expression of Mig-6, which binds and inhibits the EGFR [24]. Therefore, it has been suggested that the GC overexposure associated with PCE may upregulate Mig-6, reduce EGFR signaling, and, as a consequence, alter the EO process and bone growth [24].

Interestingly, none of these effects over EGFR were reproduced in vitro via caffeine stimulation of the primary chondrocytes [42]. Conversely, these effects were only observed once these chondrocytes were stimulated with corticosterone [24]. Thus, these data suggest that PCE exerts its deleterious effect on bone growth, at least in part, through an indirect mechanism.

#### 5.2.2. Direct and Indirect Effect of Caffeine on Growth Plate Chondrocytes

In vivo and in vitro experiments designed to evaluate the effects of caffeine on the growth plate have revealed that caffeine’s direct effects on the GPCh may be different from those observed in PCE in vivo experiments [24,27] Accordingly, caffeine’s direct effects are addressed independently in order to unravel all the potential consequences of its consumption on skeleton development and growth.

In vivo rat models of EO, developed through the subcutaneous implantation of demineralized bone particles, revealed that caffeine may exert local harmful effects on the growth plates of growing rats [34]. In these rats, caffeine ingestion reduced the expression of the c-myc oncogene, an intracellular growth promoting protein. As a result, a significant inhibition in the number of chondrocytes was observed in this experimental design [34]. Likewise, caffeine’s consumption in a dose-dependent manner decreased up to 40% of the expression of the other key growth factors and structural proteins, such as TGF-*β*, COL2A1, and the hypertrophic markers COL1A1 and OCN [34] (Figure 2). Altogether, these data highlight the fact that other GC independent mechanisms could be at play in caffeine-mediated growth plate alterations.

Consistent with the in vivo data, in vitro experiments performed on primary rat GPChs have confirmed that caffeine directly and negatively influences chondrocyte proliferation in a dose-dependent manner [27]. It was observed that caffeine alters mitochondrial integrity, affecting cell viability and promoting apoptosis [23,27,42]. These data further underpin the idea that caffeine exerts a damaging effect on the proliferation of GPChs and, therefore, on growth plate-based bone growth [27].

Considering that the ECM of the cartilage contributes to preserving chondrocyte viability, it is noteworthy that the in vitro caffeine stimulation of rat GPChs decreased the expression of cartilage-specific matrix genes, such as ACAN, COL2A1, and COLX, as well as key transcription factors involved in chondrocyte differentiation, such as Runx2 and Sox-9 [27] (Figure 2). This effect, which was also observed in articular chondrocytes [42], confirms the potential structural damage that caffeine may exert on the growth plate. The mechanism by which caffeine can alter the expression of the Runx-2 and Sox-9 transcription factors remains unclear. Nevertheless, certain studies suggest that caffeine can interfere with the second messenger cAMP, which regulates both genes [42]. As a result, caffeine’s interaction with cAMP might influence chondrocyte differentiation and maturation, which is also consistent with the alterations observed in the HZ of the growth plates of PCE rats [24,27].

Similar to the caffeine-mediated altered production of ECM components, this alkaloid also exhibits a deleterious impact on the mineralization of the growth plate [34]. It was demonstrated that caffeine reduces ALP activity in a dose-dependent manner in EO rat models and in rat GPChs [27,34]. ALP plays a critical role in cartilage matrix mineralization and is an important marker of chondrocyte maturation [27,34,42]. Thus, these results suggest that the lack of mineralization resulting from caffeine intake might be, in part, explained by its effects on ALP activity and could be related to bone growth retardation [24,34]. Consistent with the impact of caffeine on the mineralization of the growth plate [34], caffeine stimulation reduces intracellular Ca^2+^ concentration (Ca^2+^)_i_ in rat chondrocytes, which is required to achieve terminal EO [56]. The caffeine-mediated reduction of (Ca^2+^)_i_ was also linked with a diminished expression of key cartilage components, such as ACAN, COL2A1, and SPP1 [56] (Figure 2).

The mechanisms underlying the direct effects of caffeine on the growth plate have not been fully unraveled. As described above, it has been speculated that caffeine’s modulation of cAMP levels might explain certain caffeine activities [42]. Other authors have suggested that, in rat GPChs, caffeine’s effects might be partially attributed to caffeine’s binding to adenosine receptors [4]. Finally, in rat chondrocytes, caffeine’s reduction of (Ca^2+^)_i_, a process linked to catabolic processes, is mediated by its inhibition of the myo-inositol 1,4,5-triphosphate receptor [56]. All these data on caffeine’s many interactions with diverse receptors and second messengers reflect caffeine’s complex mechanism of action and highlight the elevated potential consequences of excessive caffeine consumption on growth plate cartilage [27,42,56].

## 6. Conclusions

Caffeine is one of the most commonly consumed psychostimulant substances worldwide, and its major intake sources are coffee, tea, chocolate, and soft drinks. Caffeine is a pleiotropic alkaloid that affects different organs and systems. Caffeine significantly exhibits detrimental effects on both articular and growth plate hyaline cartilage. These effects are exerted through a PCE or through its direct and indirect actions on these tissues.

Animal PCE models showed that caffeine intake, comparable to the same intake observed in some pregnant women, induces an OA-like phenotype in the articular cartilage, which is preserved into adulthood. Accordingly, caffeine also increases mechanical stress induced OA, suggesting that caffeine could be an OA promoting factor. Among the mechanisms supporting the PCE effects are the alteration of chondrocyte vitality and differentiation, the alteration of cholesterol metabolism, and the reduction of circulating and local levels of IGF-1 during fetal development. Similarly, PCE has been related to IUGR in humans and is characterized by a low birthweight, shortened bones, a retarded EO, and other growth plate cartilage abnormalities. The mechanisms underlying these effects are related to an over-exposure to the maternal GCs that affect IG-1 and EGFR signaling.

Caffeine’s direct actions have also been negatively associated with articular chondrocyte anabolism signaling, viability, proliferation, and ECM component synthesis. Likewise, caffeine may also indirectly affect the articular cartilage through the alteration of circulating levels of pro-inflammatory and anti-inflammatory cytokines. Consistent with caffeine’s direct effects on the articular chondrocytes, its direct effects on GPChs were also observed. Additionally, in GPChs, caffeine also decreases growth factor expression and interferes with their differentiation and mineralization.

Based on the data presented in this review, there is ample evidence indicating that caffeine intake negatively affects the physiology of both articular and growth plate hyaline cartilage, thereby increasing a consumer’s predisposition to suffer OA and LBGI. Due to its negative effects, caffeine consumption should be reduced and closely controlled. Specifically, this control should be mandatory for certain subjects whose caffeine metabolism is reduced, such as infants and pregnant women. As a result, health workers, such as physicians and nurses, must be aware of caffeine’s dangers to the musculoskeletal system and provide adequate advice to their patients.
